# *Bifidobacterium longum* CCFM1077 Ameliorated Neurotransmitter Disorder and Neuroinflammation Closely Linked to Regulation in the Kynurenine Pathway of Autistic-like Rats

**DOI:** 10.3390/nu14081615

**Published:** 2022-04-13

**Authors:** Qingmin Kong, Qian Chen, Xuhua Mao, Gang Wang, Jianxin Zhao, Hao Zhang, Wei Chen

**Affiliations:** 1 State Key Laboratory of Food Science and Technology, Jiangnan University, Wuxi 214122, China; 7170112017@stu.jiangnan.edu.cn (Q.K.); 18262282299@163.com (Q.C.); wanggang@jiangnan.edu.cn (G.W.); zhaojianxin@jiangnan.edu.cn (J.Z.); chenwei66@jiangnan.edu.cn (W.C.); 2 School of Food Science and Technology, Jiangnan University, Wuxi 214122, China; 3 Department of Clinical Laboratory, Yixing People’s Hospital, Wuxi 214200, China; 4 National Engineering Research Center for Functional Food, Jiangnan University, Wuxi 214122, China; 5 (Yangzhou) Institute of Food Biotechnology, Jiangnan University, Yangzhou 225004, China; 6 Wuxi Translational Medicine Research Center, Jiangsu Translational Medicine Research Institute Wuxi Branch, Wuxi 214122, China

**Keywords:** *Bifidobacterium longum*, autism, kynurenine pathway, quinolinic acid, neurotransmitter

## Abstract

The kynurenine pathway (KP) is abnormal in autistic patients and model animals. According to studies on the brain–gut axis, probiotics can help ameliorate the metabolic abnormalities of the KP in patients and model animals with neurological diseases. This study was aimed at evaluating the ability of *Bifidobacterium longum* (*B. longum*) CCFM077 to enhance the gut microbiome and KP metabolism and regulate the neurotransmitter levels and neuroinflammation of autistic rats. The KP metabolism of autistic rats was significantly disordered and significantly related to the regulation of neurotransmitter (excitation and inhibition) and neuroglia states. *B. longum* CCFM1077 could effectively alleviate autistic-like behaviours (repetitive stereotyped behaviour, learning and memory ability, and despair mood) and regulate the KP metabolism in the periphery system (gut and blood) and brain. In particular, *B. longum* CCFM1077 could significant regulate the quinolinic acid (QUIN) level in the brain and markedly regulate glutamic acid (Glu) and Glu/γ-aminobutyric acid (GABA) levels in the brain while alleviating microglia activity in the cerebellum. Through a correlation analysis, the QUIN level in the brain was strongly related with autistic-like behaviours and neurotransmitter levels (GABA and Glu). The QUIN level may thus be a potential therapeutic marker for treating autism through the intestinal and neural pathways.

## 1. Introduction

Autism spectrum disorder (ASD) is a highly heterogeneous neurological development disorder. It frequently develops in infants and young children and primarily manifests as social communication disorders, communication (verbal and non-verbal) functional impairment, and repetitive stereotyped behaviour [1]. In recent years, many researchers have suggested that gut microbiome and its metabolites can communicate with the brain bidirectionally through many elements such as immune markers, the vagus nerve, the endocrine system, and neurotransmitters [2,3,4]. Lim et al. studied the immunology and KP metabolism spectrum of ASD patients [5]. Although the serum tryptophan (TRP) concentration was similar to that of the healthy control group, the kynurenine (KYN)/TRP content for the patient group was higher, indicating the activation of KP in the patient group. The kynurenic acid (KYNA) levels of the two groups were similar; however, the serum QUIN level in the ASD group was significantly higher. This high value was the first evidence for the abnormal KP metabolism in patients with ASD [5]. In addition, patients with ASD exhibited a higher QUIN level in a urinary metabolomics analysis conducted in Italy [6]. According to genetic analyses, tryptophan 2,3-dioxygenase (IDO) polymorphism occurs in ASD. Cascio et al. detected the pathogenic variation of heavy subunit (SLC3A2) and light subunit (SLC7A5 and SLC7A8) genes by encoding large amino acid transporters (LAT) 1 and 2 in an ASD population [7]. In general, LAT 1 and 2 are responsible for the transport of tryptophan and other large aromatic amino acids through the blood–brain barrier, and they are simultaneously expressed in the blood and brain. This abnormality may affect the availability of tryptophan during brain development and may indirectly change the activity of serotonin and KP metabolism [7]. In addition, the enzyme related with tryptophan metabolism influences the regulation of genes and gene networks, which is closely related to autism [8]. ASD-related gene variations have been observed in the gene encoding NMDAr subunit, which corresponds to neural activity [9]. This evidence shows that neuroinflammation, ASD, and KP metabolism may be correlated. KP metabolism may directly or indirectly act on different neurotransmitter systems and may be influenced by several environmental conditions, such as infection, stress, and inflammation. Consequently, KP metabolism is expected to play a key role in the aetiology of ASD [10]. Many existing drugs can interfere with the critical stage of KP metabolism. Among drugs administered to patients with autism, neuroactive molecules, such as ketamine, phencyclidine, memantine, and galantamine can alleviate the symptoms of ASD by regulating glutamate and cholinergic transmission [11].

The gut microbiome has been identified as a potential key regulatory factor of KP metabolism [12]. Antibiotic-induced microbial consumption could increase the utilisation of circulating TRP and decrease the KP metabolism in the peripheral nerves [13]. Notably, the utilisation of tryptophan and KP metabolism increased in germ-free mice that recovered gut microbiome [14,15]. Therefore, it is important to enhance the KP metabolism in ASD patients and model animals. Many clinical and animal studies have been conducted using probiotics as a potential low-risk adjuvant treatment method for neurological diseases, and satisfactory experimental results have been obtained. Although these studies were limited by clinical and methodological heterogeneity, 13 comprehensive clinical studies have highlighted that probiotics could significantly influence the serum KYN and KYN/TRP ratio. These findings have provided preliminary evidence that probiotics can regulate KP metabolism [16]. In addition, *Lactobacillus plantarum* 299v can decrease the plasma KYN concentration of patients with major depression and enhance their cognitive function [17]. The consumption of *Bifidobacterium infantis* increased the concentration of TRP, decreased the metabolism from TRP to KYN, and increased the concentration of KYN in peripheral circulation in depressed rats [18]. The decrease in the serum KYN concentration of rats consuming *Lactobacillus johnsonii* is related to the ability of *L. johnsonii* in decreasing the IDO activity in HT-29 intestinal epithelial cells in vitro, likely by increasing the production of hydrogen peroxide [19]. However, the research on the regulation of KP metabolism by probiotics for treating neurological diseases has largely focused on depression, schizophrenia, and Alzheimer’s disease, and research on the regulation of KP metabolism in patients with autism or animals is limited.

To clarify the influence of Bifidobacterium on the KP metabolism of autistic rats, rats in which autistic-like symptoms were induced using valproic acid (VPA) were administered Bifidobacterium longum CCFM1077 dietary supplements from weaning to adulthood to explore their regulatory effects on autistic-like behaviours, the KP metabolism, neurotransmitter levels (excitation and inhibition), and the immune system. The findings are expected to promote the use of probiotics to treat patients with autism.

## 2. Materials and Methods

### 2.1. Animal Experiments

The pregnant Wistar rats were kept in a separate cage and divided into two groups: VPA mother group (n = 10) and CON mother group (n = 5). At E 12.5, the pregnant rats were intraperitoneally injected with 600 mg/kg sodium VPA (mixed in normal saline) in the VPA mother group and same dose of normal saline in the control (CON) mother group [20]. All born rats were randomly assigned to the mother rats (rats were covered up strange odours with new mother rats’ bedding to prevent infanticide), and the number of rats in per litter was consistent to avoid litter effects to eliminate the different from offspring growth and maternal behaviour during the lactation period. Autistic-like symptoms have been reported to be more pronounced in males, in both animals and humans; therefore, male offspring were selected for further experiments in the present study. On postnatal day 21, 18 male rats born in the VPA mother group were randomly allocated to the VPA-reduced group (autistic-like rats), while 6 male rats born in the CON mother group were randomly allocated to in the CON group (healthy rats).

From 4 to 8 weeks of age, 6 autistic-like rats were gavaged with *B. longum* CCFM1077 at 10^9^ CFU/mL (CCFM1077 group), 6 autistic-like rats were gavaged with normal saline (VPA group), 6 autistic-like rats were gavaged with risperidone (MED group, 1.6 μg/100 g, mixed in normal saline), and 6 healthy rats were gavaged with normal saline (CON group). Details of the *B. longum* CCFM1077 cell suspension in normal saline are presented in Supplementary Material Table S1 After the behaviour tests, blood and tissues were collected and frozen at −80 °C. The study was approved by the Ethics Committee of Experimental Animals of Jiangnan University, China (JN. no. 20180915S0601230(183)). The procedures were performed following the European Community Guidelines for the Care and Use of Experimental Animals (directive 98 2010/63/EU).

### 2.2. Behavioural Tests

The behavioural tests included open-field test (OFT), marble-burying test (MBT), social test (ST), Y-maze test (YMT), and forced swim test (FST) [21,22,23]. The detail information is presented in the Supplementary Materials.

### 2.3. Neuroendocrine Secretion Analysis

The 50 mg freeze-dried faeces, 100 μL serum, and 50 mg brain tissue were used in the analysis of the levels of Trp, KYN, KYNA, and QUIN; 50 mg brain tissue was used in the analysis of the levels of Glu, GABA, norepinephrine (NE), and acetylcholine (ACH). The detailed materials and methods are provided in the Supplementary Materials [24,25].

### 2.4. Immunohistochemical Analysis

Detailed materials and methods of the protein expression (*IBA-1* and *GFAP*) are provided in the Supplementary Materials [26].

### 2.5. Gut Microbiota Analysis

DNA of faeces (50 mg) was collected after behaviour tests using the FastDNA^®^ Spin Kit for Feces. The detailed materials and methods are found in the Supplementary Materials [27].

### 2.6. Statistical Analyses

Statistical analyses were assessed as means ± standard deviations (SD) and plotted as means with 95% confidence intervals using IBM SPSS Statistics 23 and GraphPad Prism 8.0.2. Unpaired Student’s *t*-test was performed between the CON and VPA groups. The significance of differences among the other groups compared with the VPA group was evaluated using a one-way analysis of variance (ANOVA) followed by Dunnett’s multiple comparisons test. Asterisks in the figures represent the following: * *p* < 0.05; ** *p* < 0.01; *** *p* < 0.001. The detailed statistical analyses are found in the Supplementary Materials.

## 3. Results

In this study, it was found that KP metabolism was strongly related with the neurotransmitter (excitation and inhibition) regulation and neuroglial status. *B. longum* CCFM1077 could significantly regulate the QUIN, Glu, and Glu/GABA levels in the brain and ameliorate the microglia activity in the cerebellum. The ability of *B. longum* CCFM1077 in alleviating autistic-like behaviours (repetitive stereotyped behaviour, learning and memory ability, and despair mood) through the KP pathway was attributable to the enhancement in the QUIN and neurotransmitter levels.

### 3.1. B. longum CCFM1077 Could Alleviate Autistic-like Behaviors

**Figure 1:**
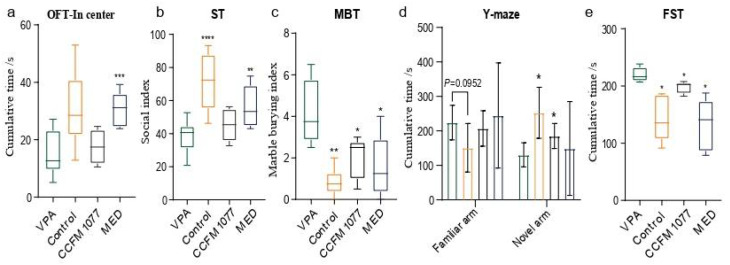
*B. longum* CCFM1077’s effect on alleviating autistic-like behaviours. (**a**) Open filed test (OFT): The accumulated time in the centre zone. (**b**) Social test (ST): The social index. (**c**) Marble burying test (MBT): The number of marbles buried. (**d**) Y-maze test (YMT): The accumulated time in the novel arm. (**e**) Force swimming test (FST): The accumulated time of stopping and floating. Unpaired Student’s *t*-test was performed between the CON and VPA groups. Data are mean with SD (n = 5–6 per group for each test). ANOVA following by Dunnett’s multiple comparisons test performed against the VPA group. Asterisks in the figures represent the following: * *p* < 0.05; ** *p* < 0.01; *** *p* < 0.001; **** *p* < 0.0001.

Rats in which autistic-like symptoms were induced using VPA exhibited significant behavioural abnormalities (Figure 1a–e). Compared with the healthy group, autistic rats exhibited significantly decreased exploratory behaviour in unfamiliar environments (*p* < 0.0001, Figure 1a), impairments in social interaction (*p* < 0.0001, Figure 1b), repetitive stereotyped behaviour (*p* = 0.0019, Figure 1c), and learning and memory ability impairments (*p* = 0.0103 for the cumulative time in a novel arm, Figure 1d), along with despair mood (*p* = 0.0111, Figure 1e). *B. longum* CCFM1077 was also effective in ameliorating autistic-like behaviours, in particular, repetitive stereotyped behaviour (*p* = 0.0213, Figure 1c), learning and memory ability impairments (*p* = 0.0414 for the cumulative time in the novel arm, Figure 1d), and despair mood (*p* = 0.0165, Figure 1e). Risperidone, which has been noted to be beneficial in the treatment of children and adolescents with ASD, could significantly alleviate the autistic-like behaviours of VPA-induced autistic-like rats.

### 3.2. Effect of B. longum CCFM1077 on KP Metabolism Was Related to Autistic-like Behaviours

**Figure 2:**
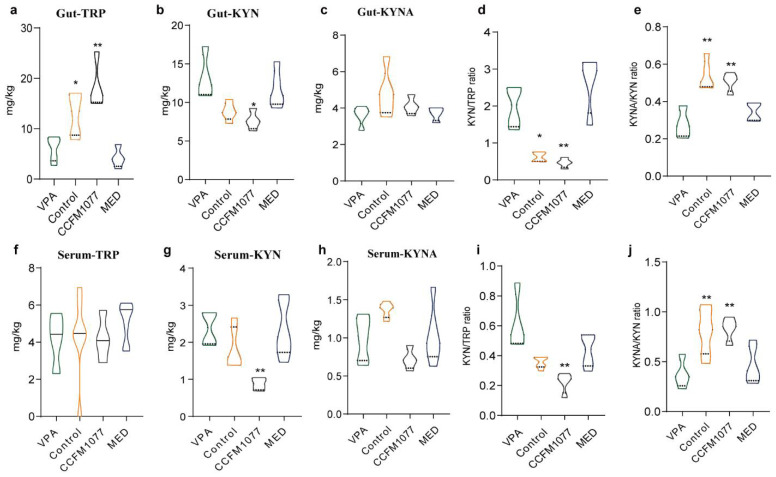

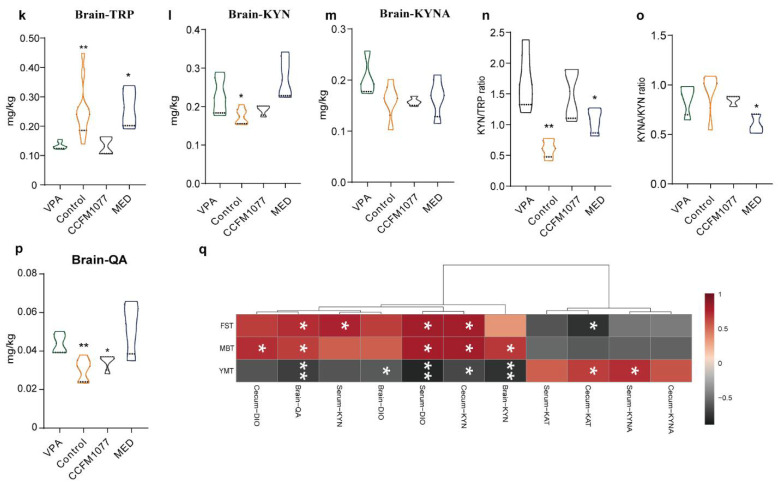
*B. longum CCFM1077*’s effect on KP metabolites and neurotransmission in serum and brain. (**a**–**c**) The Trp, KYN, and KYNA levels in the caecum. (**d**) The ratio of KYN/Trp in the caecum. (**e**) The ratio of KYNA/KYN in the caecum. (**f**–**h**) The Trp, KYN, and KYNA levels in the serum. (**i**) The ratio of KYN/Trp in the serum. (**j**) The ratio of KYNA/KYN in the serum. (**k**–**m**) The Trp, KYN, and KYNA levels in the brain. (**n**) The ratio of KYN/Trp in the brain. (**o**) The ratio of KYNA/KYN in the brain. (**p**) The QA level in the brain. (**q**) The correlation analysis between behaviours and the KP metabolite in the caecum, serum, and brain. Data are mean with SD (n = 5–6 per group for each test). Unpaired Student’s *t*-test was performed between the CON and VPA groups. ANOVA following by Dunnett’s multiple comparisons test performed against the VPA group. Asterisks in the figures represent the following: * *p* < 0.05; ** *p* < 0.01.

Compared with the healthy rats, the levels of TRP and main metabolites of KP in the cecum, serum, and brain of autistic rats were abnormal. In the cecum, the TRP level was significantly lower (*p* = 0.0121, Figure 2a, and the KYN (*p* = 0.0505, Figure 2b) and KYN/TRP (*p* = 0.0135, Figure 2d) levels were significantly higher for the autistic rats than for the healthy rats. The KYNA levels of both groups were similar (Figure 2c), but the KYNA/KYN value for autistic rats was significantly lower (*p* = 0.0016, Figure 2e). In the serum, the TRP, KYN, and KYNA levels were similar for both groups; however, the KYNA/KYN level was significantly lower (*p* = 0.0065, Figure 2j) for autistic rats. The same abnormality as that in the cecum was also observed in the brain (Figure 2l–p). In the case of autistic rats treated with *B. longum* CCFM1077, the TRP level in the cecum was upregulated (*p* = 0.0040, Figure 2a), the KYN level in the cecum significantly decreased (*p* = 0.0119, Figure 2b), the KYN/TRP level significantly decreased (*p* < 0.0011, Figure 2d), and the KYNA/KYN level significantly increased (*p* = 0.0008, Figure 2e), suggesting that *B. longum* CCFM1077 influences the KYN level in the serum by regulating it from the gut. Moreover, the KYN (*p* = 0.0044, Figure 2g), KYN/TRP (*p* = 0.0022, Figure 2i), and KYNA/KYN levels (*p* = 0.0029, Figure 2j) in the serum of autistic-like rats treated with *B. longum* CCFM1077 were normalised. In addition, *B. longum* CCFM1077 decreased the KYN level in the brain (*p* = 0.3465, Figure 2l), albeit non-significantly. Moreover, *B. longum* CCFM1077 significantly decreased the QUIN level in the brain (*p* = 0.0103, Figure 2). Risperidone could not regulate the KP metabolism. According to a correlation analysis (Figure 2q), repetitive stereotyped behaviour, learning and memory ability impairments, and despair mood were strongly correlated with the metabolite in the KP, especially the peripheral KYN level and QUIN level in the brain. Therefore, in the context of KP metabolism, *B. longum* CCFM1077 might alleviate the autistic-like behaviours through the QUIN level in the brain.

### 3.3. Regulation Effect of B. longum CCFM1077 on the KP Metabolism Was Related to Gut Microbiome Enhancement

**Figure 3:**
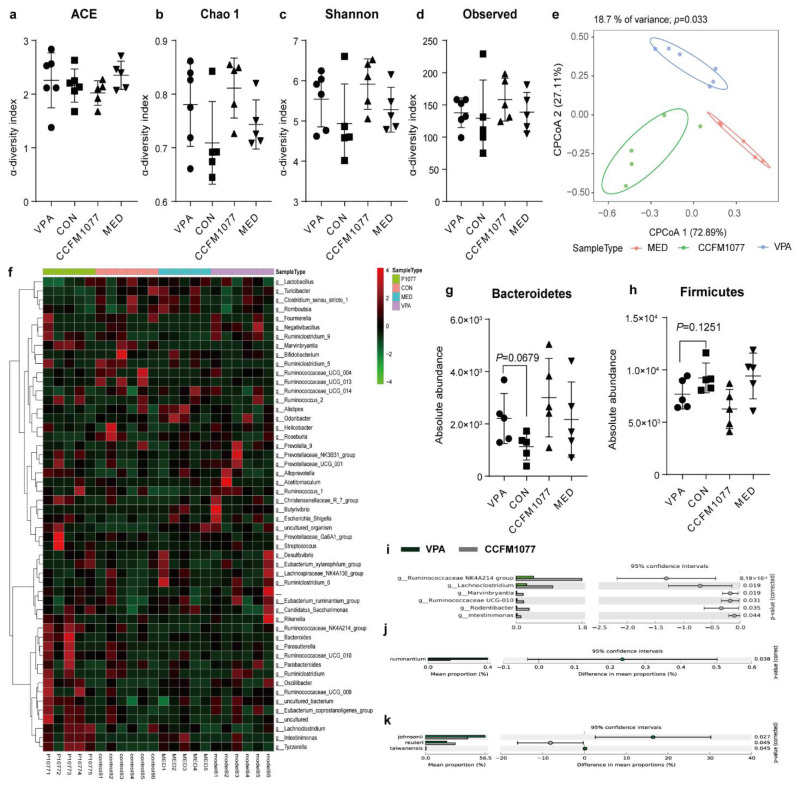
*B. longum* CCFM1077’s effect on gut microbiome. (**a**–**d**) The α-diversity evaluated using the ACE, Chao1 Shannon, and observed indices. (**e**) Beta diversity reflected by PCoA based on unweighted UniFrac distances of the gut microbiome. (**f**) The heatmap of the gut microbiome on the level of genus. (**g**) The abundance of Bacteroidetes. (**h**) The abundance of Firmicutes. (**i**) The different gut microbiomes on the gene level between the groups VPA and *B. longum* CCFM1077. (**j**) The different gut microbiomes on the level of Bifidobacterium between the groups VPA and *B. longum* CCFM1077. (**k**) The different gut microbiomes on the level of Lactobacillus between the groups VPA and *B. longum* CCFM1077. The data are mean with SD (n = 5–6 per group for each test). Unpaired Student’s *t*-test was performed between the CON and VPA groups. ANOVA following by Dunnett’s multiple comparisons test performed against the VPA group. Asterisks in the figures represent the following.

*B. longum* CCFM1077 did not considerably regulate the α diversity (Figure 3a–d). On the basis of the Bray–Curtis distance, the differences among four groups were analysed through CPCOA (Figure 3e). The maximum total variation for the VPA, CCFM1077, and MED groups was 18.7%, and a significant difference was observed (*p* < 0.033). The heat map of the genus level based on the cluster analysis between groups showed that the distribution of the genus level in the group treated with *B. longum* CCFM1077 was similar to that of the healthy rat group (Figure 3f). Moreover, *B. longum* CCFM1077 did not considerably regulate the level of Bacteroidetes (*p* = 0.3477, Figure 3g) and Firmicutes (*p* = 0.2157, Figure 3h) in autistic-like rats. On the gene level (Figure 3i), *B. longum* CCFM1077 significantly increased the abundance of the *Ruminococcaceae NK4A214 group* (*p =* 0.0082) and *Lachnoclostridium* (*p =* 0.019), and significantly decreased the abundance of *Ruminococcus UCG-010* (*p =* 0.031), *Intestinimonas* (*p =* 0.044), *Rodentibacter* (*p =* 0.035), and *Marvinbryantia* (*p =* 0.019). At the *Bifidobacterium* level (Figure 3j), *B. longum* CCFM1077 significantly decreased the *B. ruminantium* abundance (*p =* 0.038). Moreover, *B. longum* CCFM1077 influenced the abundance of *B. Pseudomonas subsp. Globosum*, *B. animalis* (*B. animalis subsp. animalis*), and *B. longum* (*B. longum subsp. infantis* and *B. longum subsp. longum*) (Figure S1a–f), but this influence was not notable. On the level of *Lactobacillus* (Figure 3k), *B. longum* CCFM1077 significantly decreased the abundance of *L. johnsonii* (*p =* 0.027), *L. reuteri* (*p =* 0.045), and *L. taiwanensis* (*p =* 0.045) and regulated the abundance of *L. intestinalis*, *L. mucosae*, *L. murinus*, and *L. acidophilus* (Figure S1h–k). The abundance of the *Ruminococcaceae NK4A214* group was strongly correlated with the KYNA and KYNA/KYN levels in the serum. The abundance of *Ruminococcaceae UCG-010* was negatively correlated with the KYNA level in the serum, and that of *L. johnsonii* was positively correlated with the KYN level in the brain. The abundance of *L. reuteri* was negatively and positively correlated with the KYN and KYNA levels in the serum, respectively. The abundance of *L. taiwanensis* was negatively correlated with the KYNA/KYN level in the serum, and that of *B. pseudocatenulatum* was positively correlated with the KYN/TRP and KYN levels in the cecum and brain. The abundance of *L. murinus* was negatively correlated with the KYN level in the brain. Thus, the regulation of KP metabolism by *B. longum* CCFM1077 was closely related to the corresponding enhancement in the gut microbiome.

### 3.4. B. longum CCFM1077 Could Likely Regulate Neurotransmitters Levels by Promoting the KP Metabolism

**Figure 4:**
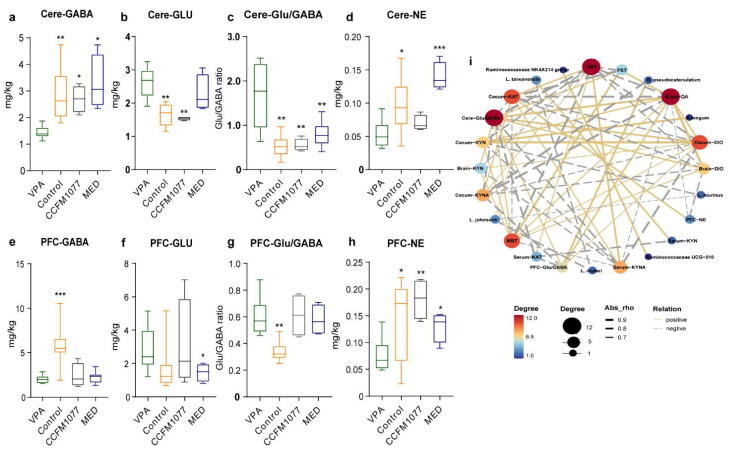
*B. longum* CCFM1077’s effect on the level of neurotransmitters. (**a**) The level of GABA in the cerebellum. (**b**) The level of Glu in the cerebellum. (**c**) The ratio of Glu and GABA in the cerebellum. (**d**) The level of NE in the cerebellum. (**e**) The level of GABA in the prefrontal cortex. (**f**) The level of Glu in the prefrontal cortex. (**g**) The ratio of Glu and GABA in the prefrontal cortex. (**h**) The level of NE in the prefrontal cortex. (**i**) The interaction network analysis. The positive and negative relation were performed with the yellow and grey line, respectively. The correlation coefficient was in the Supplementary Materials Table S2. DIO: KYN/TRP; KAT: KYNA/KYN. Data are mean with SD (n = 5–6 per group for each test). Unpaired Student’s *t*-test was performed between the CON and VPA groups. ANOVA followed by Dunnett’s multiple comparisons test was performed against the VPA group. Asterisks in the figures represent the following: * *p* < 0.05; ** *p* < 0.01; *** *p* < 0.001.

The levels of GABA and NE in the cerebellum of autistic rats were significantly lower and those of Glu and Glu/GAGA were significantly higher than those of healthy rats (Figure 4a–d), whereas the DA levels for both groups were similar (Figure S1l,m). The changes in the other neurotransmitters in the prefrontal cortex (PFC), except Glu (Figure 4e–h and S1n,o), were identical to those in the cerebellum. *B. longum* CCFM1077 considerably influenced the GABA (*p =* 0.0109, Figure 4a), Glu (*p =* 0.0069, Figure 4b), and Glu/GABA (*p =* 0.0024, Figure 4c) levels in the cerebellum and NE in the PFC (*p =* 0.0051, Figure 4h). The inhibitory and excitatory neurotransmitter levels were strongly correlated with the metabolite levels in KP, and the Glu and Glu/GABA levels in the cerebellum and PFC were strongly correlated with the QUIN level in the brain. Moreover, risperidone could significantly regulate the GABA, NE, and Glu/GABA levels in the cerebellum and Glu and NE levels in the PFC. According to the interaction network analysis (Figure 4i), the autistic-like behaviours (repetitive stereotyped behaviour, learning and memory ability, and despair mood) were influenced by the gut microbiota, QUIN, and neurotransmitter (excitation and inhibition) levels.

### 3.5. B. longum CCFM1077 Could Ameliorate the Microglial Activity

**Figure 5:**
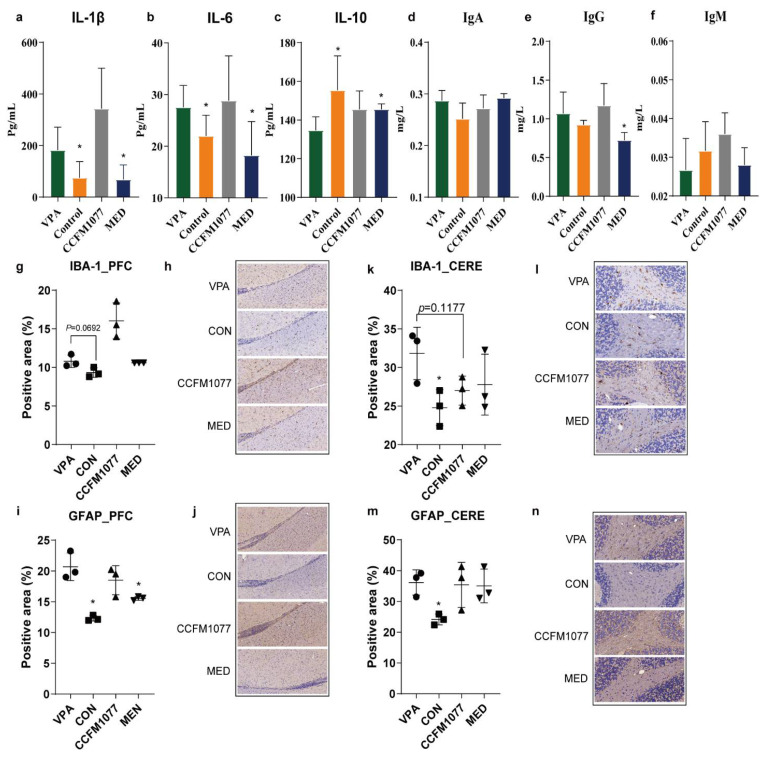
*B. longum* CCFM1077’s effect on immune system. (**a**–**f**) The level of IL-1β, IL-6, IL-10, IgA, IgG, and IgM in the serum. (**g**–**j**) The protein expression of *IBA-1* and *GFAP* in the prefrontal cortex. (**k**–**n**) The protein expression of *IBA-1* and *GFAP* in the cerebellum. Data are mean with SD (n = 5–6 per group for immune factor in the serum; n = 3 per group for immunohistochemical analysis). Unpaired Student’s *t*-test was performed between the CON and VPA groups. ANOVA followed by Dunnett’s multiple comparisons test was performed against the VPA group. Asterisks in the figures represent the following: * *p* < 0.05.

The autistic rats exhibited chronic inflammation. The levels of proinflammatory factors (IL-1β and IL-6) in the serum increased (*p =* 0.0393, Figure 5a; *p =* 0.0432, Figure 5b) and that of anti-inflammatory factor IL-10 decreased (*p =* 0.0354, Figure 5c). The levels of immunoglobins G, A, and M (IgG, IgA, and IgM, respectively) remained unchanged (Figure 4c–e). The use of risperidone could regulate the levels of proinflammatory factors IL-1β and IL-6 in the serum and slightly increase the level of IL-10. However, when the rats were treated with *B. longum* CCFM1077, the levels of proinflammatory factors and IgA in the serum of autistic-like rats were not regulated, and the anti-inflammatory factor IL-10 level was negligibly increased.

Moreover, significant abnormalities were observed in the microglia and astrocyte activities in the PFC (*p =* 0.0487, Figure 5g,h; *p =* 0.0202, Figure 5i,j) and cerebellum (*p =* 0.0692, Figure 5k,l; *p* = 0.0234, Figure 5m,n) of autistic rats. *B. longum* CCFM1077 could ameliorate the microglia activity in the cerebellum (*p =* 0.1177, Figure 5k,l), and risperidone could significantly enhance the activation of astrocytes in the PFC (*p =* 0.0285, Figure 5i,j). According to the relevance analysis, the neuroglia activities were related to the QUIN level in the brain. Thus, *B. longum* CCFM1077 ameliorated neuroinflammation and was related to the regulation of KP metabolism of autistic-like rats.

## 4. Discussion

Both autistic patients and model animals exhibit abnormal KP metabolism, attributable to the abnormal tryptophan metabolism caused by gut microbiome disorders [5,11,12,16]. Probiotics can likely promote tryptophan metabolism by regulating the intestinal environment, thereby enhancing the behaviours and mood [28]. Research on the KP regulation of probiotics to treat neurological diseases has mostly been focused on depression, schizophrenia, and Alzheimer’s disease [16,29], and research on the regulation of KP metabolism in autistic people or animals remains limited. In this study, to explore the regulatory effects of probiotics on the KP metabolism of patients with autism, rats in which autistic-like symptoms were induced using VPA were administered *B. longum* CCFM1077 dietary supplements from weaning to adulthood. The analysis revealed that KP metabolism was strongly related with the neurotransmitter (excitation and inhibition) regulation and neuroglial status. *B. longum* CCFM1077 could significantly regulate the QUIN, Glu, and Glu/GABA levels in the brain and ameliorate the microglia activity in the cerebellum. The ability of *B. longum* CCFM1077 in alleviating autistic-like behaviours (repetitive stereotyped behaviour, learning and memory ability, and despair mood) through the KP pathway was attributable to the enhancement in the QUIN and neurotransmitter levels.

TRP, as the main neuromodulator between the gut and brain, has attracted considerable research attention. TRP is considered the core of the complex network between immune and neuroendocrine systems because it is involved in many physiological and pathological processes, such as nerve transmission, oxidative stress, and immune responses [30]. According to research on TRP consumption in human beings, decrease in the TRP availability affects the attention of situational memory and memory of language information [28,30]. For adults with ASD, tryptophan deprivation in the diet has been noted to aggravate the symptoms of autism [31]. Therefore, the metabolic balance of tryptophan in autistic patients or animals may be the key to alleviate autistic-like symptoms. KP is the main pathway of TRP catabolism, and many studies have indicated that the KP pathway is associated with neurodevelopmental disorders owing to its involvement in neuroprotection and immune regulation [10,32]. Moreover, the levels of tryptophan, KYN, and KYNA in the blood of children with autism are low, and the QUIN concentration is significantly high [5,6,11]. Moreover, the levels of KYN and KYNA in the periphery (gut and blood) of VPA-induced autistic-like rats are significantly low. Therefore, VPA-induced autistic-like rats can simulate the abnormal KP metabolic pathway of humans with autism.

Autistic-like behaviours are prominent in patients with autism, aged approximately two years [33], and the glutamic acid content is often significantly high, which may be associated with excitotoxic neuron damage and/or neuronal connection damage [34,35]. Children with ASD have ‘noisy’ and unstable cortical networks, likely because of the disproportionately high levels of excitatory neurotransmitters or disproportionately weak inhibitory neurotransmitters [36]. QUIN is considered to play a central role in glutamate excitotoxicity, synaptic plasticity changes, neural network oscillation abnormalities, seizures and epilepsy, learning and memory disorders, visual system abnormalities, dyskinesia, behavioural changes, and social dysfunction [37,38,39], which are commonly observed in patients with ASD. According to the findings of this study, the main abnormalities of neurotransmitters in the cerebellum and prefrontal cortex of autistic rats corresponded to GABA/Glu, and *B. longum* CCFM1077 could significantly regulate the GABA/Glu level in the cerebellum, which is closely related with the QUIN level in the brain. Chai et al. suggested that QUIN and other neuroactive KP metabolites can regulate the glutamate activity and limit excitotoxicity [40,41]. The use of NMDA antagonists, such as memantine, has long been proposed as an adjuvant therapy for ASD [42]. Studies have shown that memantine treatment can weaken the poly (ADP-ribose) polymerase (PARP) activity of human primary neurons induced by QUIN, and memantine can significantly enhance the emotional functions and behaviours [43]. Patients with depression and schizophrenia have been noted to exhibit high QUIN levels and intense microglia activity [44,45]. Steiner et al. highlighted that the microglia activity was associated with elevated the QUIN level in the cingulate cortex subregion of patients with severe depression [44]. These regions are also affected in ASD patients and exhibit a high density of functional NMDA and AMPA receptors [46,47]. Notably, certain researchers have introduced the virus poly (I:C) in pregnant mice to stimulate elevated the QUIN level and schizophrenia-like behaviours in the offspring [48,49]. These aspects indicate that schizophrenia and autism may share neuro-pathogenic mechanisms. It is speculated that schizophrenia is related to the excessive inhibition of glutamate system by KYNA, whereas autism can be attributed to the abnormal QUIN level [49]. In this study, the KYNA levels in the peripheral blood and brain of autistic and healthy rats were similar, and the QUIN levels in the brain were significantly different. These observations revealed that the significant abnormality of the QUIN levels in the brains of autistic rats is the key factor for the change in the related physiological indexes. Consequently, several studies have recommended the use of specific KP enzyme inhibitors to manipulate the KP pathway to decrease the QUIN level to limit the progress and/or severity of ASD [50,51]. Therefore, it is reasonable to use QUIN as a potential therapeutic target for probiotics to regulate KP metabolism and alleviate autistic-like behaviours in patients.

The antiepileptic effect of low doses (relative 600 mg/kg) of VPA is associated with increasing GABA in the brain [52]. Conversely, in this study, VPA-treated rats showed a lower concentration of GABA in the cerebellum and prefrontal cortex. This could be explained by the fact that the effect of a high-dose intraperitoneal injection of VPA on embryos at 12.5 days of pregnancy mainly prevents the closure of the embryonic neural tube, causing an embryonic spinal cord nerve defect [53,54], then affecting the structure and function of the cortex, cerebellum, brain stem, and so on [55,56]. It is these early hypomorphic developments that trigger the autistic-like behavioural phenotype. The effect of this short-term high-dose intraperitoneal injection of VPA on embryos is very different from the inhibition of GABA transferase activity caused by continuous low-dose VPA administration. Moreover, in a study conducted in 2013, the KYN concentration in the hippocampus region was noted to rapidly increase after VPA i.p. injection [57]. Moreover, the TRP level in the plasma gradually decreased, and the KYN level in the peripheral blood first decreased slightly and then increased. Although VPA re-entered the centre from the vein, the KP in the brain was activated first. This initial activation likely occurred because the VPA stimulated the KP metabolism; large amounts of TRP and KYN were derived from the periphery, which eventually was saturated, and thus the peripheral KP metabolism exhibited abnormalities [57]. In this case, the abnormal KP metabolism in the brain directly drove the peripheral KP metabolism. It remains to be known, however, whether the KP metabolism in the brain was dominant, or the metabolism processes in the periphery and brain were synchronised. In this study, no direct stimulus of VPA was provided. After VPA induction in the second trimester of pregnancy, autistic rats exhibited the abnormal state. The intervention of *B. longum* CCFM1077 might be reversed in comparison with the direct stimulation process of the VPA. Specifically, *B. longum* CCFM1077 first enhanced the KP metabolism in the gut, and then affected the peripheral blood, ultimately enhancing the KP metabolism in the brain. Once the intervention of *B. longum* CCFM1077 was terminated, it is unknown as to whether the abnormal KP metabolism in brain continues to interfere with the KP metabolism in the periphery. Thus, it is also necessary to prove the elution stage of *B. Longum* CCFM1077 administration.

The potential of probiotic use as a low-risk auxiliary intervention technique has been proven by limited clinical studies, according to which probiotics can regulate the KP metabolic activity in the disease state by decreasing the KYN and KYN/TRP levels in the serum. The abnormal manifestations of the KP metabolism in different diseases may have different emphases [16]. The present study confirms the speculation of previous studies. The abnormal KP metabolism in patients with autism is closely related to the QUIN level in the brain. Therefore, we have the reason to speculate that the use of probiotics can enhance the QUIN metabolism, instead of the KYNA level, in the brain by regulating the KP metabolism in the periphery system and brain, thereby alleviating autistic-like behaviours. Nevertheless, this study involves certain limitations. Because of the limited detection means, we only evaluated the KYN, KYNA, and QUIN in the KP metabolism. The effect of intervention of probiotics on intermediate metabolites such as 3HK and 3HIAA need to be examined in future work.

## 5. Conclusions

KP metabolism interferes with the course of many neurological diseases and is considered one of the causes of ASD. This study found that KP metabolism is strongly related to the neurotransmitter (excitation and inhibition) regulation and neuroglia status. *B. longum* CCFM1077 could significantly regulate QUIN, Glu, and Glu/GABA levels in the brain and ameliorate the microglia activity in the cerebellum. The ability of *B. longum* CCFM1077 in alleviating autistic-like behaviours (repetitive stereotyped behaviour, learning and memory ability, and despair mood) through the KP pathway could be attributed to the increase in the QUIN and neurotransmitter levels. Given the promising role of *B. longum* CCFM1077 in modulating autistic behaviours by regulating the KP metabolism, future studies must focus on the corresponding molecular mechanism.

## Data Availability

The datasets generated and analysed during the current study are available from the corresponding author on reasonable request.

## Supplementary Materials

The following supporting information can be downloaded at https://www.mdpi.com/article/10.3390/nu14081615/s1. Additional methods; Table S1: The ingredients of the intervention product. Table S2: Correlation coefficient. Figure S1: *B. longum* CCFM1077’s effect on gut microbiome and neurotransmitters.
