# Supplementary material for: Bifidobacterium longum CCFM1077 Ameliorated Neurotransmitter Disorder and Neuroinflammation Closely Linked to Regulation in the Kynurenine Pathway of Autistic-like Rats
Source: Nutrients. 2022 Apr 13;14(8):1615. doi: 10.3390/nu14081615 (PMC9031594; doi:10.3390/nu14081615)
Supplement: Supplementary file 1 [file nutrients-14-01615-s001.zip › Supplementary Information.pdf]

Supplementary Information

Bifidobacterium longum **CCFM1077 Ameliorated Neurotransmitter Disorder and Neuroinflammation Closely Linked to Regulation in the Kynurenine Pathway of Autistic-like Rats**
Kong et al Suppl.

Table of Contents

Additional methods 2

Table S1 The ingredients of the intervention product 4

Figure S1 ***Bifidobacterium longum CCFM1077*****'s effect on gut microbiome and neurotransmitters.** 4

Additional methods

1. Behavioural tests

1.1 Open-field test

An open-field box of 40 × 40 × 100 cm (length × width × height) with blackened inner walls was used for the open-field test. EthoVision software (EthoVision Pro, Noldus Inc., Leesburg, VA) was used to calculate the time spent at the center. The field of vision of the camera covered the entire interior area of the open-field box. The observation time for each rat was 6 min [21].

1.2 Marble-burying test

The marble-burying test was performed in a rat cage of 42 × 26 × 15 cm (length × width × height). The cage was covered with a 5 cm layer of a corncob. Twenty marbles were distributed throughout the cage. The rats were kept in the cage containing the marbles for 30 min. Following this, the marble burying response was quantified by counting the number of marbles that were buried more than two-thirds in the bedding materials [21].

1.3 Social test

The social test was performed in a three-chambered cage of 100 × 100 × 60 cm (length × width × height). The experimental device consisted of three rectangular boxes, each with a specification of 100 × 30 × 60 cm (length × width × height). The partition between each box was made of transparent plexiform glass, and there was a channel in the middle to connect the three boxes (chambers). A day before the experiment, all the selected rats were placed in the middle chamber to adapt to the three chambers. On the next day (during the experiment), a test rat was placed in the middle chamber and the channels leading to the left and right chambers were closed for 10 min to allow the rat to adapt to the middle chamber. An empty wire cage was then placed in the left chamber, and a strange rat was placed in the right chamber and covered with a wire cage. The channels leading from the middle chamber to the left and right chambers were opened, and the behavior of the test rat was recorded for 10 min using a video camera. The social index was defined as the ratio of the residence time in the right chamber (strange rat) of the cage to the total residence time in the left and right chamber of the cage and was calculated using EthoVision software [21].

1.4 Y-maze test

Y-maze is shaped as a Y, consisting of three similar arms that are all placed at 120 degrees to each other.Measurement of spatial memory using the Y-maze with a blocked arm (novel arm). Rat rats first adapted to two open arms for 10 min. On the next day, the blocked arm was open, the rats were placed in three similar open arms. The rat with good spatial memory will enter the previously novel arm more frequently than the other arms [22].

1.5Forced swimming test

In forced swim test, the time spent swimming versus the time spent floating in a tall cylinder (height, 40 cm; diameter, 20 cm) filled with water was measured. This physical immobility was considered an indication of behavioral despair. The accumulated time when swimming was stopped and floating was started was calculated using EthoVision software and used to assess the rats' desperation during the monitoring period of 6 min [23].

2. Metabolite and neurotransmitters analysis

The caecum contain weighing 50 mg , serum measuring 100 μL and brain weighing 50 mg were respectively homogenized with 900 μL cold MeOH/H2O (1:1), 800 μL cold MeOH, and 900 μL cold MeOH/H2O (1:1), vortexed for 15 s, placed at -20 °C for 1h, then centrifugated at 12000×g for 15 min. The supernatant (900 μL) was concentrated under vacuum (Thermo Scientific, USA) at 45 °C for 2 h. The resultant dry residues were reconstituted in 100 μL of MeOH/H2O (1:9) and transferred to chromatographic sample bottles for analysis.

An HPLC system (Waters, USA) coupled with a TSQ Quantum XLS triple-quadrupole mass spectrometer (Waters, USA), equipped with an electrospray ion source, and operated in the positive ion mode was used. The aliquot (2 μL) was injected with mobile phases A (0.1% formic acid in water) and B (acetonitrile) using a ACQUITY UPLC® BEH C18 1.7 μm (150 mm×2.10 mm) column to analyze the metabolite levels (L-Trp, kynurenine (KYN), kynurenic acid (KYNA), quinolinic acid (QUIN)) [24] and the neurotransmitters level (glutamic acid (Glu), γ-aminobutyric acid (GABA), norepinephrine (NE), acetylcholine (AcH)) [25]. All the standard substances were purchased from Sigma-Aldrich.

3. Immunohistochemistry analysis

Fresh colon and brain tissues were isolated from rat, washed with PBS, fixed with 4% formaldehyde solution and embedded in paraffin. Colon and brain paraffin sections were heated at 60°C for 60 min and de-waxed in xylene boiling. Antigen retrieval was carried in water bath antigen retrieval in citric acid buffer (pH=6.0) for 15min. After washing three times for 5 min each in phosphate buffered saline Tween 20 (PBST), They were incubated overnight with monoclonal IBA-1 antibody (1:500, Abcam, Cat#ab5076) / GFAP antibody (1:800, Millipore, Cat#MAB360, then rewarmed for 40 min at room temperature; For immunohistochemistry, each slices were incubated with 50 μl horseradish peroxidase (HRP) secondary antibodies for 45 min at room temperature, after washing, incubated with the peroxidase or fluorescent-conjugated secondary antibodies for 1 h in room temperature. Then tissue sections were successively stained by DAB work solution, hematoxylin. Mount the slides with permount TM mounting medium. Cover the slides with coverslips. Sections were scanned using a

high-resolution digital scanner to collect images and analyzed with Image pro plus 6.0 [26].

4. Gut microbiota analysis

DNA from the fecal samples of the rats in the groups above were collected on the 56th day after birth using the FastDNA® Spin Kit for Feces (MP Biomedicals, Santa Ana, CA, USA) following the manufacturer's instructions. The V3-V4 region of 16S rRNA was amplified with universal primers (341F CCTAYGGGRBGCASCAG and 806R GGACTACNNGGGTATCTAAT). The products were purified using the TIANgel Mini Purification Kit (TIANGEN, Beijing, China) and quantified using the Qubit dsDNA HS Assay Kit (Life Technologies Corporation, Carlsbad, CA, USA). Pair-end reads with an overlap of >10 bp and without mismatches were assembled. The operational taxonomic unit (OTU) was established de novo using UCLUST with a 97% similarity threshold. The first sequence in each OTU cluster was chosen as the representative sequence and then aligned to the SLIVA core set in QIIME 2 using the PyNAST aligner [27].

5. Statistical analyses

Statistical analyses were performed using SPSS 20.0 and GraphPad Prism 7. The data were assessed for normality of distribution and are presented as means ± standard deviations (SD) and plotted as means with 95% confidence intervals. Unpaired Student's t-test was performed between the CON and VPA groups. The significance of differences among the other groups compared with the VPA group was evaluated using a one-way analysis of variance (ANOVA) following by Dunnett's multiple comparisons test. Asterisks in the figures represent the following: *P<0.05; **P<0.01; ***P<0.001. Alpha (α) diversity assessment and beta (β) diversity assessment (using constrained PCoA (CPCoA) of the gut microbiota) were performed using R software. Differential gut microbiota is presented using STAMP. Spearman correlation coefficients between behaviors, OTU abundance, neurotransmitters and metabolite were present in Heatmap and network analysis.

Table S1 The ingredients of the intervention product

Table S1 The ingredients of the intervention product

**Probiotic type**	**Source**	**Dosage** mL/100g (weight)
B. longum CCFM1077	dairy product	10^9 CFU/mL (NS)

Figure S1 *Bifidobacterium longum CCFM1077*'s effect on gut microbiome and neurotransmitters.

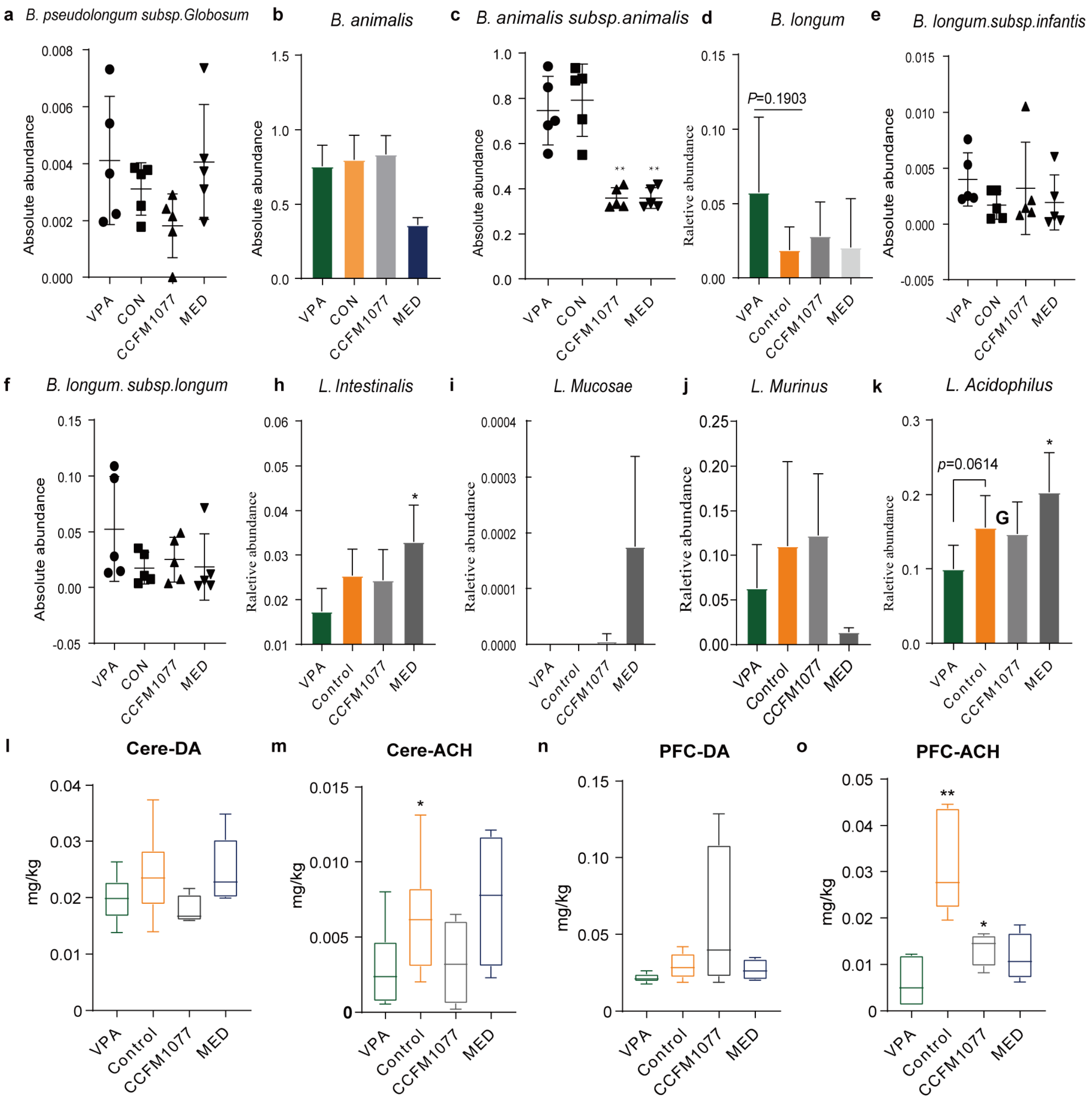

(**a-f**) *B. longum CCFM1077* effect on the *Bifidobacterium*. (**h-k**) *B. longum CCFM1077* effect on the *Lactobacillus*. **L.** *B. longum CCFM1077* effect on the level of DA in cerebellum. (**m**) *B. longum CCFM1077* effect on the level of ACH in cerebellum. (**n**) *B. longum CCFM1077* effect on the level of DA in prefrontal cortex. (**o**) *B. longum CCFM1077* effect on the level of ACH in prefrontal cortex. Unpaired Student's t-test was performed between the CON and VPA groups. The significance of differences among the other groups compared with the VPA group was evaluated using a one-way analysis of variance (ANOVA) following by Dunnett's multiple comparisons test. Asterisks in the figures represent the following: **P*<0.05; ***P*<0.01. The detailed statistical analyses are in supplemental files.
